# Root PRR7 Improves the Accuracy of the Shoot Circadian Clock through Nutrient Transport

**DOI:** 10.1093/pcp/pcad003

**Published:** 2023-01-07

**Authors:** Kyohei Uemoto, Fumito Mori, Shota Yamauchi, Akane Kubota, Nozomu Takahashi, Haruki Egashira, Yumi Kunimoto, Takashi Araki, Atsushi Takemiya, Hiroshi Ito, Motomu Endo

**Affiliations:** Division of Integrated Life Science, Graduate School of Biostudies, Kyoto University, Yoshida-Konoecho, Sakyo-ku, Kyoto, 606-8501 Japan; Graduate School of Science and Technology, Nara Institute of Science and Technology, 8916-5 Takayama-Cho, Ikoma, Nara, 630-0192 Japan; Faculty of Design, Kyushu University, 4-9-1 Shiobaru, Minami-ku, Fukuoka, 815-8540 Japan; Department of Biology, Graduate School of Sciences and Technology for Innovation, Yamaguchi University, 1677-1 Yoshida, Yamaguchi, 753-8512 Japan; Graduate School of Science and Technology, Nara Institute of Science and Technology, 8916-5 Takayama-Cho, Ikoma, Nara, 630-0192 Japan; Graduate School of Science and Technology, Nara Institute of Science and Technology, 8916-5 Takayama-Cho, Ikoma, Nara, 630-0192 Japan; Graduate School of Science and Technology, Nara Institute of Science and Technology, 8916-5 Takayama-Cho, Ikoma, Nara, 630-0192 Japan; Graduate School of Science and Technology, Nara Institute of Science and Technology, 8916-5 Takayama-Cho, Ikoma, Nara, 630-0192 Japan; Division of Integrated Life Science, Graduate School of Biostudies, Kyoto University, Yoshida-Konoecho, Sakyo-ku, Kyoto, 606-8501 Japan; Department of Biology, Graduate School of Sciences and Technology for Innovation, Yamaguchi University, 1677-1 Yoshida, Yamaguchi, 753-8512 Japan; Faculty of Design, Kyushu University, 4-9-1 Shiobaru, Minami-ku, Fukuoka, 815-8540 Japan; Graduate School of Science and Technology, Nara Institute of Science and Technology, 8916-5 Takayama-Cho, Ikoma, Nara, 630-0192 Japan

**Keywords:** Circadian clock, Mathematical model, Micrografting, Potassium, Root-to-shoot signaling

## Abstract

The circadian clock allows plants to anticipate and adapt to periodic environmental changes. Organ- and tissue-specific properties of the circadian clock and shoot-to-root circadian signaling have been reported. While this long-distance signaling is thought to coordinate physiological functions across tissues, little is known about the feedback regulation of the root clock on the shoot clock in the hierarchical circadian network. Here, we show that the plant circadian clock conveys circadian information between shoots and roots through sucrose and K^+^. We also demonstrate that K^+^ transport from roots suppresses the variance of period length in shoots and then improves the accuracy of the shoot circadian clock. Sucrose measurements and qPCR showed that root sucrose accumulation was regulated by the circadian clock. Furthermore, root circadian clock genes, including *PSEUDO-RESPONSE REGULATOR7* (*PRR7*), were regulated by sucrose, suggesting the involvement of sucrose from the shoot in the regulation of root clock gene expression. Therefore, we performed time-series measurements of xylem sap and micrografting experiments using *prr7* mutants and showed that root PRR7 regulates K^+^ transport and suppresses variance of period length in the shoot. Our modeling analysis supports the idea that root-to-shoot signaling contributes to the precision of the shoot circadian clock. We performed micrografting experiments that illustrated how root PRR7 plays key roles in maintaining the accuracy of shoot circadian rhythms. We thus present a novel directional signaling pathway for circadian information from roots to shoots and propose that plants modulate physiological events in a timely manner through various timekeeping mechanisms.

## Introduction

The plant circadian clock regulates the expression of thousands of genes involved in various physiological processes such as photosynthesis and nutrient uptake, thereby enhancing plant fitness in fluctuating environments (Harmer et al. 2000, Dodd et al. 2005, Haydon et al. 2015). Plant circadian clocks are based on an intracellular transcriptional–translational feedback loop among the clock genes *CIRCADIAN CLOCK ASSOCIATED1* (*CCA1*), *LATE ELONGATED HYPOCOTYL* (*LHY*), *TIMING OF CAB2 EXPRESSION1* (*TOC1*) and *PSEUDO-RESPONSE REGULATOR* (*PRR*). In addition, the evening complex, composed of LUX ARRHYTHMO (LUX), EARLY FLOWERING 3 (ELF3) and ELF4, and REVEILLE form another feedback loop with the PRR protein family (Davis et al. 2022). While each plant cell shows robust circadian rhythmicity, circadian parameters such as period length and phase can differ substantially among plant organs (Thain et al. 2002, James et al. 2008, Yakir et al. 2011, Takahashi et al. 2015, Bordage et al. 2016, Endo 2016, Gould et al. 2018). These differences would reduce the synchrony of circadian rhythms among tissues, making it difficult for the plant to respond to environmental fluctuations in a coordinated manner. In mammals, circadian rhythms are governed by a central oscillator residing in the suprachiasmatic nucleus, which controls circadian rhythms in peripheral organs. In plants, a hierarchical communication framework has been proposed, wherein the root clock relies on a long-distance signal from the shoot apex (Takahashi et al., 2015), and at the same time the leaf tissues contribute to a decentralized network architecture (Shimizu et al., 2015). To achieve coherent and synchronized circadian oscillations across the plant body, plant circadian clocks communicate circadian information between cells and tissues. Several studies have reported that temporal information is transferred through cell-to-cell, tissue-to-tissue and organs-to-organ communication in plants (Wenden et al. 2012, Endo et al. 2014, Takahashi et al. 2015, Greenwood et al. 2019, Chen et al. 2020). Cell-to-cell coupling has been observed in leaves and roots (Wenden et al. 2012, Greenwood et al. 2019). At the tissue level, vascular cells regulate the circadian rhythms of neighboring mesophyll cells (Endo et al. 2014). At the organ level, sucrose and ELF4 have been proposed to be long-distance signaling of temporal information, as they are transported from shoots to roots and regulate the period length of circadian rhythms (Takahashi et al. 2015, Chen et al. 2020). However, most of the long-distance signals that have been identified so far are from the shoot cells, and the existence and role of long-distance signaling originating from the root circadian clock are unclear.

Whole-plant analysis has revealed that sucrose represses the expression of *PRR7* and maintains a proper circadian period length (Haydon et al. 2013, Frank et al. 2018). Since sucrose is a major metabolite transported to roots through the vascular bundle, it was proposed to contribute to shoot-to-root signaling for circadian information; however, the precise mechanism is not understood. Besides sucrose, several cations were also transported across the plant body and were reported to affect the circadian clock. For example, the macronutrients magnesium (Mg^2+^) and calcium (Ca^2+^) affect the circadian clock. Magnesium nutrition regulates the pace of the circadian clock, likely by affecting global transcription/translation (de Melo et al. 2021, Rivière et al. 2021). The disruption of cytosolic Ca^2+^ concentration also resulted in longer period lengths, suggesting that cytosolic Ca^2+^ signaling is involved in modulating the circadian clock (Martí Ruiz et al. 2018). By contrast, no study has indicated that K^+^, which is another major cation, affects the circadian clock. However, recent studies have suggested that K^+^ acts as a signaling molecule, making K^+^ a potential signal component for root-to-shoot communication for circadian information (Rubio et al. 2014, Brauer et al. 2016, Shabala 2017), which would be consistent with the transport via the vascular bundle to reach shoots.

Although plant circadian clocks in individual cells communicate to achieve a synchronized rhythm, long-distance signaling of circadian information is much less understood. Here, we focused on sucrose and K^+^ cations as signal components between shoots and roots and validate their involvement in circadian communication. Sucrose quantification revealed that sucrose accumulation in roots exhibits circadian oscillations. Furthermore, the variation in the expression of clock genes in response to sucrose and 3-(3,4-dichlorophenyl)-1,1-dimethylurea (DCMU) tended to be greater in roots than in shoots, indicating that the root circadian clock is highly sensitive to sucrose. These results suggest that the circadian oscillation of sucrose might regulate the root circadian rhythm through the regulation of the expression of clock genes such as PRR7 in roots. To test the function of PRR7 in roots, time-series analysis of xylem sap showed that the cation concentration in xylem sap was significantly altered in mutants and overexpressors of PRR7, suggesting that root PRR7 is involved in root-to-shoot cation transport, including K^+^. Furthermore, since disruption of K^+^ transport increases the variability of the shoot circadian cycle, we hypothesized that K^+^ transport contributes to maintaining the accuracy of the shoot circadian clock. Therefore, to test whether root PRR7 improves the accuracy of the shoot period length, we performed micrografting experiments and found that functional inhibition of root PRR7 increased shoot period length variability. These results suggest that root PRR7 contributes to the accuracy of the shoot circadian clock by regulating K^+^ transport. Our results propose the novel concept that the accuracy of the circadian clock is maintained by a rhythm maintenance mechanism mediated by inter-organ communication, which contributes to stable rhythmic progression even in fluctuating environments.

## Results

### Circadian oscillations of sucrose in roots correlate with the expression pattern of root PRR7

**Fig. 1:**
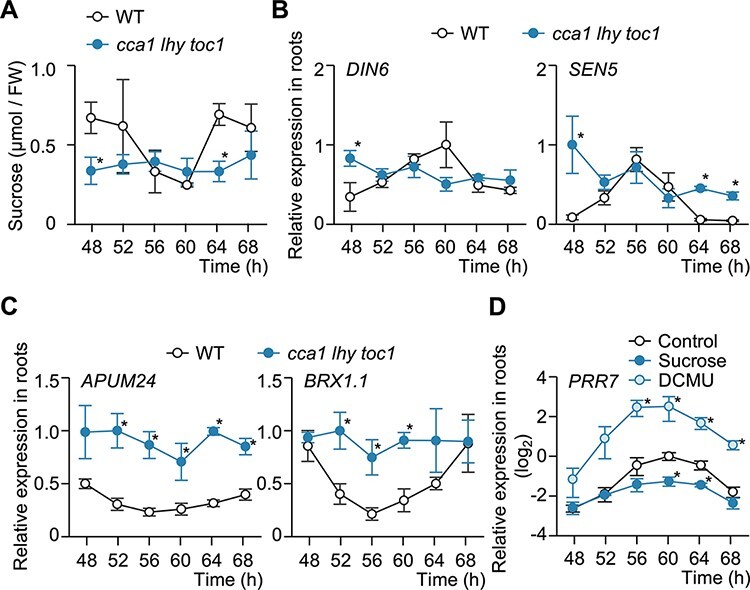
Root *PRR7* is likely regulated by circadian oscillations of sucrose accumulation in roots. (A) Rhythms in sucrose accumulation in WT (Col-0) and *cca1 lhy toc1* roots in LL (*n* = 3). (B, C) Time course of relative expression levels for the sucrose-repressed genes (*DIN6* and *SEN5*) (B) and the sucrose-induced genes (*APUM24* and *BRX1-1*) (C) in WT and *cca1 lhy toc1* roots by RT-qPCR analysis (*n* = 3). (D) Time course of *PRR7* in WT roots with exogenous sucrose or DCMU treatment by RT-qPCR analysis (*n* = 3). All growth media did not contain sucrose except under sucrose conditions. Data are mean ± standard error of the mean (SEM). *P < 0.05 compared to the WT or control; two-sided Student’s *t*-test (A–C) or Dunnett’s test (D).

Sucrose has been proposed as a temporal signal for shoot-to-root communication (Haydon et al. 2013, Takahashi et al. 2015, Frank et al. 2018). To elucidate the effect of the sucrose signal from shoots, we explored the effect of sucrose transport on the root circadian system. To this end, we measured sucrose levels in roots under circadian conditions [constant light (LL)] following entrainment under light–dark (LD) cycles. Sucrose accumulation in Col-0 [wild type (WT)] roots showed an oscillation pattern, with a trough around subjective dusk at time 60 with the last dark-to-light transition being time 0; by contrast, sucrose levels failed to display a rhythmic pattern in the arrhythmic mutant *cca1 lhy toc1* (**Fig. 1A**). This result suggested that the circadian clock regulates sucrose accumulation in roots. As roots are nonphotosynthetic organs, sucrose accumulation may be regulated by the shoot circadian clock.

To investigate the effect of sucrose levels on gene expression in roots, we monitored the circadian expression pattern of sugar-responsive genes in roots. The sucrose-repressed genes *DARK INDUCIBLE6* (*DIN6*) and *SENESCENCE*-*ASSOCIATED PROTEIN5* (*SEN5*) (Baena-González et al. 2007, Shin et al. 2017) reached their highest expression levels out of phase with that of sucrose in WT roots (**Fig. 1B**). Conversely, the expression of the sucrose-inducible genes *ARABIDOPSIS PUMILIO PROTEIN24* (*APUM24*) and *BIOGENESIS OF RIBOSOMES IN XENOPUS1-1* (*BRX1-1*) (Maekawa et al. 2018) was in phase with sucrose accumulation in WT roots (**Fig. 1C**). Importantly, the expression pattern of all four genes was disrupted in *cca1 lhy toc1* roots (**Fig. 1B, C**). These results suggest that sucrose oscillation in roots affects the rhythmicity of sucrose-responsive gene expression in this organ.

To examine the effect of sucrose accumulation on the root circadian clock, we monitored the expression of clock genes in roots treated with the photosynthesis inhibitor DCMU or with exogenously applied sucrose 2 d before transferring into LL. Similar to its expression in shoots (Haydon et al. 2013), *PRR7* expression in roots peaked around dusk (time 60) and was more highly expressed in DCMU-treated seedlings but expressed at lower levels upon exogenous sucrose treatment (**Fig. 1D**, **Supplementary Fig. S1A**). Expression of *PRR5* and *PRR9* in roots showed a similar response to DCMU and exogenous sucrose as *PRR7*, but their responsiveness was lower than that of *PRR7* (**Supplementary Fig. S1A**). Responsiveness to sucrose and DCMU for other clock genes was also examined in shoots and roots, but all genes examined had milder responses than PRR7 in roots (**Supplementary Fig. S1A, B**).

### Root PRR7 is involved in the regulation of cation transport from roots to shoots

**Fig. 2:**
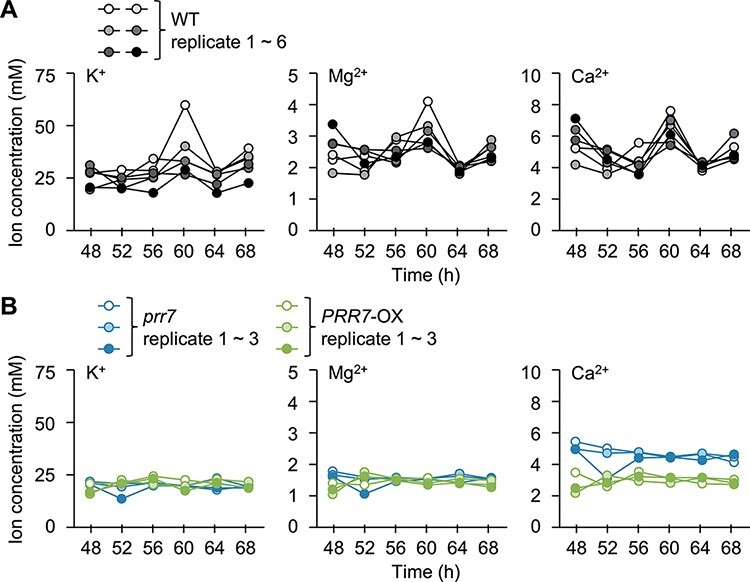
Root *PRR7* is involved in the pattern of cation concentrations in the xylem sap. (A, B) K^+^, Mg^2+^ and Ca^2+^ cation concentrations of xylem sap in the WT (A) or *prr7* and *PRR7-*OX (B) released into LL over one circadian cycle (WT; *n* = 6; *prr7* and *PRR7*-OX; *n* = 3). After the removal of leaves, xylem sap was collected from the hypocotyl stump of 30 plants for 1 h.

These whole-seedling analyses indicate that PRR7 is involved in the regulation of the circadian clock through sucrose signaling. Since the root circadian clock regulates a variety of physiological processes, including nutrient transport (Haydon et al. 2015), we next investigated the role of root PRR7 in root-to-shoot nutrient transport. Time-series analysis of the major cations K^+^, Mg^2+^ and Ca^2+^ in xylem sap showed that the concentrations of these ions changed over time in the WT, but in the *prr7* mutant and PRR7-overexpressing (PRR7-OX) seedlings, neither cation concentration changed and their concentrations were nearly constant at all times (**Fig. 2**).

Cation concentration in xylem sap is affected not only by uptake but also by water flow through transpiration, and previous studies have reported that PRR7 is involved in transpiration (Liu et al. 2013) Therefore, we further confirmed whether root PRR7 contributes to cation transport by calculating the amount of cations transported to shoots from cation concentrations in xylem sap and water flow. The amount of transported cations was reduced in *prr7*, and even *PRR7*-OX differed significantly from the WT (**Supplementary Fig. S2B**). While it remains possible that the effects of transpiration are mitigated in these lines, the possibility that root PRR7 is involved in root-to-shoot cation transport is indicated.

### Disruption of K^+^ transport impairs the accuracy of the shoot circadian clock

**Fig. 3:**
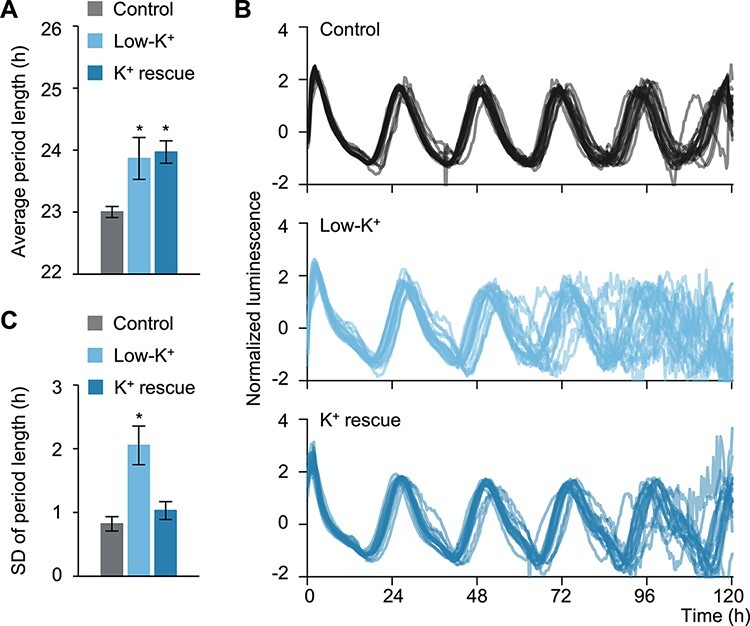
Low-K^+^ conditions disturb K^+^ transport to shoots and decrease the accuracy of period length in shoots. Circadian oscillations of *LHYpro:LUC* under control, low-K^+^ or K^+^ rescue conditions in LL (*n* = 20). (A) Average period length, as calculated for each plant across the 4 d in LL. (B) Waveforms of individual *LHYpro:LUC* bioluminescence. Waveforms were detrended and compensated for decreasing amplitude over the time course. (C) SD of period length, as calculated for each plant across the 4 d in LL. Data are mean ± SEM. * *P* < 0.05 compared to control by Dunnett’s test.

We explored the role of cation transport in regulating the shoot circadian clock by measuring the bioluminescence of seedlings harboring a transgene consisting of the firefly *LUCIFERASE* (*LUC*) reporter gene driven by the *LHY* promoter (*LHYpro:LUC*), whose bioluminescence signal is mainly derived from the aerial parts of seedlings (**Supplementary Fig. S3**). We monitored *LHYpro:LUC* bioluminescence under cation deficiency conditions (low K^+^, low Mg^2+^ and low Ca^2+^), in which we presumed cation transport to be disturbed. As determined by the mean period length of *LHYpro:LUC* rhythms, although the effect of low-Ca^2+^ conditions was small, the period length increased under these three conditions (**Fig. 3A**, **Supplementary Fig. S4A**). The long period length caused by low Mg^2+^ was consistent with previous reports (de Melo et al. 2021, Rivière et al. 2021). Interestingly, only low-K^+^ medium significantly diminished the synchrony of shoot circadian rhythms in individual seedlings, as evidenced by the gradual loss of synchrony between seedlings (**Fig. 3B**, **Supplementary Fig. S4B, C**). To better assess the accuracy of period length, we calculated the period length in four 24-h windows over the 96-h time course and determined the associated standard deviation (SD) for individual seedlings. We observed that the SD of period length significantly increases in response to low K^+^ (**Fig. 3C**). Moreover, to test whether K^+^ transport could suppress the escalation of SD, we performed a K^+^ rescue experiment. To avoid the exposure of shoots to KCl, we covered roots with a sterilized sheet and added KCl under the sheets. While K^+^ resupply did not rescue the longer period length seen under low-K^+^ conditions, SD returned to a value similar to that obtained under control conditions (**Fig. 3B, C**), suggesting that K^+^ transport is involved in maintaining the accuracy in shoots.

These results suggest that K^+^ transport contributes to the accuracy of period length in shoots; however, since K^+^ has pleiotropic functions, we cannot rule out the possibility that this phenotype is indirectly related to K^+^ starvation, rather than an impairment of K^+^ transport. We therefore compared the growth of WT seedlings under our control and low-K^+^ conditions. The size and color of seedlings were identical between the two conditions, indicating that the low accuracy of period length in shoots is not caused by severe growth defects (**Supplementary Fig. S5**). Also, K^+^ starvation attenuates photosynthetic activity, which prompted us to test the effect of exogenous sucrose (2%, w/v) under low-K^+^ conditions on circadian precision. Notably, exogenous sucrose did not neutralize the effect of low K^+^ on the circadian rhythms of the *LHYpro:LUC* reporter in shoots (**Supplementary Fig. S6**). We also tested the effect of K^+^ excess by characterizing the *LHYpro:LUC* pattern in the shoots of seedlings exposed to high-K^+^ conditions. Importantly, high-K^+^ concentrations also increased the SD of period length in shoots (**Supplementary Fig. S7**). These results suggest that the low accuracy of period length in shoots is due to the disturbance of K^+^ transport, not K^+^ starvation.

Low circadian amplitude leads to a weaker robustness of circadian rhythms (Masuda et al. 2017), suggesting the possibility that lower amplitude may result in the loss of synchrony of circadian rhythms. Indeed, the amplitude decreased under low-K^+^ conditions (**Supplementary Fig. S8**). However, we showed that K^+^ resupply does not compensate for the effect of low K^+^ on the amplitude or average period length (**Fig. 3A**, **Supplementary Fig. S8**). Considering that K^+^ resupply improved the accuracy of period length (**Fig. 3B, C**), this result suggests that the disturbance of K^+^ transport impairs the accuracy of period length without affecting the amplitude or average period length. Together, our results indicate that K^+^ transport, not K^+^ availability, contributes to the accuracy of the shoot circadian clock.

### A two-oscillator model can explain the contribution of root-to-shoot signaling for the accuracy of the shoot circadian clock

**Fig. 4:**
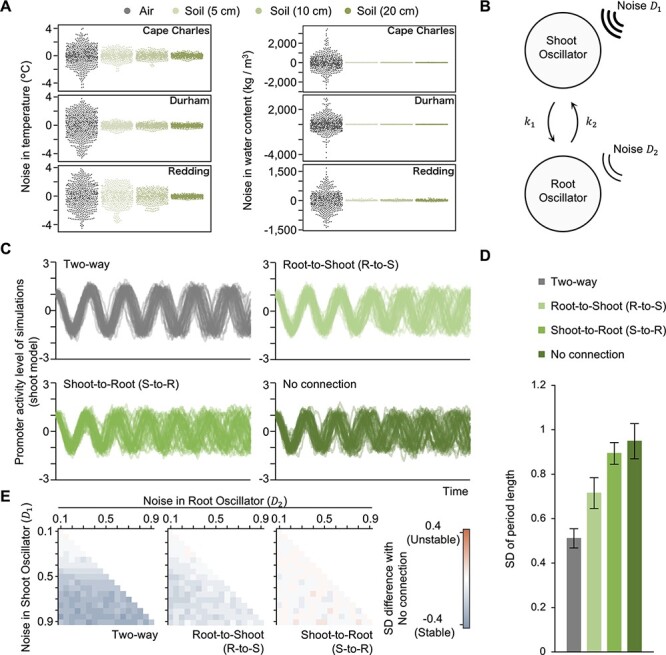
Mathematical modeling predicts that root-to-shoot signaling contributes to the accuracy of period length in shoots under fluctuating conditions. (A) Estimation of noise at ambient and soil temperatures in three cities in the USA (Cape Charles, Durham and Redding). The noise is a ‘remainder component’, that is, the residuals from the seasonal plus trend fitting of temperature and moisture, as analyzed by STL (Cape Charles, *n* = 576; Durham, *n* = 624; Redding; *n* = 528). (B) Overview of the mathematical model used in the simulation. The shoot oscillator and root oscillator are autonomous oscillators coupled with a given connection strength (*k*_1_, *k*_2_) and are independently affected by environmental noise (*D*_1_, *D*_2_). The average period length and amplitude in the shoot oscillator are longer and larger than those in the root oscillator, respectively. For more information, see the Materials and methods section. (C, D) Simulated waveforms of the shoot oscillator when it is connected to the root oscillator (two-way), when one oscillator is dominant over the other (R-to-S and S-to-R), and when the oscillators are not coupled (no connection), with the average SD of period length in the shoot oscillator (*n* = 50). Data are mean ± SEM. (E) Difference in SD for period length in the shoot oscillator compared to ‘no connection’ in each condition when the magnitude of noise varied from 0.1 to 0.9 and *D*_1_ is higher than *D*_2_ (*D*_1_ > *D*_2_).

We provided evidence for a shoot-to-root signal (sucrose) and a root-to-shoot signal (K^+^ transport) that improve the accuracy of shoot circadian rhythms. To validate the effect of these signals on the accuracy of the circadian system, we performed a modeling analysis. Previous studies have employed a simplified mathematical model of the circadian clock in plants, but a more accurate model should consider that (i) the characteristics of the shoot and root circadian clocks are different (Greenwood et al. 2019, Chen et al. 2020), (ii) the strength of their connections may not be the same (Takahashi et al. 2015) and (iii) the intensity of noise affecting circadian rhythms might be different between tissues. In fact, we established that roots and shoots are not exposed to the same amplitude of seasonal cues, based on the calculation of the remainder (noise), which refers to the remaining component after periodic components and trends were removed by the Seasonal Trend–decomposition procedure based on Loess (STL) from field weather data of temperature and moisture, with leaves being subjected to more noise than roots (**Fig. 4A**, **Supplementary Fig. S9**).

We employed a two heterogeneous oscillator–based model with independent coupling strength and environmental noise (see the Materials and methods section) (**Fig. 4B**). We performed 50 independent trials and estimated the contribution of signaling to the accuracy of rhythms. The SD of period length in the shoot oscillator decreased when the shoot oscillator was entrained by the root oscillator (R-to-S) or when the two oscillators communicated (two-way) (**Fig. 4C, D**). On the contrary, the SD of period length in the shoot oscillator did not change when the root-to-shoot signaling was shut down (S-to-R). These results suggest that root-to-shoot signaling contributes to the accuracy of the shoot oscillator. Furthermore, we performed a grid search for noise parameters under conditions that produce more noise in shoots than in roots (*D*_1_ > *D*_2_). Over a wide range of noise parameters, the SD of period length in the shoot oscillator increased under both R-to-S and two-way conditions (**Fig. 4E**). We also performed a grid search for the other parameters, connection strength and time lag. Previous micrografting experiments showed that the rhythm of the root circadian clock is synchronized with that of the shoot circadian clock (Takahashi et al. 2015). Moreover, long-distance transport between shoots and roots takes at least 10–60 min in *Arabidopsis* (Knox et al. 2018, Endo et al. 2019). Considering these studies, we performed a grid search of connection strength in *k*_1_ > *k*_2_ and time lag in the range of 0–3 h. Under both R-to-S and two-way conditions, the SD of period length in the shoot oscillator declined compared to a situation with no connection (**Supplementary Fig. S10**), supporting our observations that the root circadian clock contributes to the accuracy of the shoot circadian clock. Interestingly, the accuracy of the shoot oscillator showed a greater improvement under two-way conditions than under other conditions tested here (**Fig. 4C****–****E**, **Supplementary Fig. S10**), suggesting that plants might form shoot–root coupling for a more precise circadian rhythm. Taken together, our simulation confirms that the root-to-shoot signaling between circadian clocks can reduce the effect of noise over various conditions, suggesting that the root circadian clock might work as a stabilizer for the shoot circadian clock under natural conditions.

### Root PRR7 contributes to the accuracy of the shoot circadian clock

**Fig. 5:**
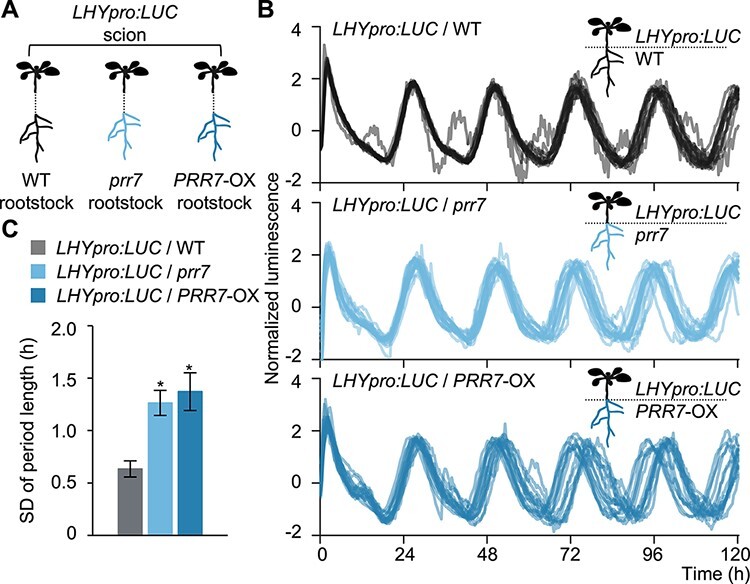
Root PRR7 improves the accuracy of period length in shoots. (A) Schematic diagram of the LUC assay conducted on micrografted plants. *LHYpro:LUC* scions were grafted onto WT (*LHYpro:LUC*/WT), *prr7* (*LHYpro:LUC*/*prr7*) or *PRR7-*OX (*LHYpro:LUC*/*PRR7*-OX) rootstocks. (B, C) Individual *LHYpro:LUC* bioluminescence traces (B) and SD of period length under LL (*n* = 15) (C). Waveforms were detrended and compensated for the decrease in amplitude over the time course. The SD values were calculated for each plant across 4 d in LL. Data are mean ± SEM. *P < 0.05 compared to *LHYpro:LUC/*WT by Dunnett’s test.

Our results suggested that the root circadian clock, especially root PRR7, improves the accuracy of period length in shoots by regulating K^+^ transport. To elucidate the contribution of root PRR7 directly, we grafted *LHYpro:LUC* scions (in the WT background) onto WT, *prr7* and *PRR7*-OX rootstocks missing the reporter gene (**Fig. 5A**). While WT rootstocks had a small SD for period length in shoots, both *prr7* and *PRR7*-OX rootstocks showed a high SD for period length in the WT scions (**Fig. 5B, C**), suggesting that root PRR7 contributes to the accuracy of period length in shoots. However, neither *prr7* nor *PRR7*-OX rootstocks caused obvious effects on the amplitude or average period length (**Supplementary Fig. S11**). Since K^+^ transport improved the accuracy of the shoot circadian clock without affecting the amplitude or average period length (**Fig. 3**, **Supplementary Fig. S8**), we conclude that the low accuracy induced by *prr7* and *PRR7*-OX rootstocks can likely be attributed to the disturbance of K^+^ transport. In addition, the missing and overexpression of PRR7 affected the pattern of K^+^ transport without decreasing K^+^ transport severely (**Fig. 2**). Considering this result, our results suggest that root PRR7 contributes to the accuracy of the shoot circadian clock probably by regulating rhythmic K^+^ transport.

## Discussion

Roots send nutrient-related signals to shoots and modulate the expression of downstream genes responsible for nutrient transport. For example, the C-TERMINALLY ENCODED PEPTIDE family and *trans*-zeatin-type cytokinin convey nitrogen-deficiency signals to shoots (Kiba et al. 2013, Ko et al. 2014, Tabata et al. 2014). Strigolactone and aminocyclopropane-1-carboxylic acid function as signals for inorganic phosphate and K deficiency, respectively (Kohlen et al. 2011, Martínez-Andújar et al. 2016). Here, we suggested that rhythmic K^+^ uptake itself functions as a root-to-shoot signal that affects the circadian clock in shoots (**Figs. 2**, **3**, **5**). The circadian regulation of K^+^ uptake or transport has been observed in various organisms (Kondo and Tsudzuki 1978, Sato et al. 1985, Feeney et al. 2016). In duckweed (*Lemna gibba*), K^+^ uptake in roots exhibits a circadian rhythm, and the K^+^ channel blocker affects the circadian period length (Kondo and Tsudzuki 1978, Kondo 1990). Moreover, K^+^ is thought to function as a signaling molecule (Rubio et al. 2014, Brauer et al. 2016, Shabala 2017). These observations support our suggestion that rhythmic K^+^ transport acts as a root-to-shoot signal. Although we do not know how the relatively small changes in K^+^ concentrations affect the circadian clock, our modeling of the circadian clock system in shoots and roots supported the idea that root-to-shoot signaling improves the accuracy of period length by affecting the phase in shoots (**Fig. 4**). Considering this result, K^+^ signaling might modulate the phase of the shoot circadian clock to improve its accuracy. Experiments that transiently disturb K^+^ transport will be helpful to understand the molecular mechanism by which the K^+^ input feeds into the circadian clock.

Whole-plant analyses have demonstrated that the expression level of *PRR7*, probably in shoots, is regulated by sucrose (Haydon et al. 2013, Frank et al. 2018). Considering that sucrose is transported to roots, sucrose is thought to convey the temporal information of shoots to roots by a largely unknown mechanism. Here, we determined that sucrose accumulation in roots follows a circadian oscillation and that root *PRR7* expression levels are affected in response to sucrose (**Fig. 1**). The sucrose in roots is likely coming from the shoot and will reflect the activity of the shoot circadian clock. These results suggest that the rhythmic sucrose transport from shoots conveys the temporal information to roots by regulating root *PRR7* expression. Besides root *PRR7*, root *PRR5* and *PRR9* also exhibited sucrose-responsive expression. In addition, root *CCA1* expression also increased upon DCMU treatment (**Supplementary Fig. S1**). By contrast, whole-plant analyses showed that, although PRR7 in shoots is regulated by sucrose, neither *CCA1, PRR5* nor *PRR9* exhibits a similar sucrose responsiveness in shoots (Haydon et al. 2013). This difference in sucrose responsiveness between shoots and roots likely reflects the organ specificity of the circadian clock system. Indeed, *OsCCA1* in rice (*Oryza sativa*) displays a different response to sucrose in roots and shoots (Wang et al. 2020). Compared to shoots, roots may be less exposed to strong environmental fluctuations (**Supplementary Fig. S9**). High sucrose responsiveness might enable the root circadian clock to acquire the temporal information from shoots more acutely.

Our time-series analysis of xylem sap indicated that cation transport is regulated by root PRR7 (**Fig. 2**, **Supplementary Fig. S2**). PRRs are partially redundant, as single *prr* mutants have weak phenotypes in many cases (Farré et al. 2005, Nakamichi et al. 2005, Salomé and McClung 2005). Nevertheless, cation transport is clearly affected in *prr7* (**Fig. 2**, **Supplementary Fig. S2**), suggesting that root PRR7 might modulate nutrient transport specifically. PRRs regulate their downstream genes with other transcription factors (Nakamichi et al. 2012, Liu et al. 2016, Hayama et al. 2017, Zhang et al. 2020). The specific function of root PRR7 in cation transport might be due to an organ-specific interaction with another transcription factor. We described here a novel function for root PRR7 that had remained hidden in whole-plant analyses. It is of great interest to discover the organ-specific function of PRR7.

In many cases, to elucidate circadian rhythms, the average from several individual seedlings is plotted and analyzed. In this study, we focused on the accuracy of period length by measuring the variation of period length in a single seedling under LL, which revealed the contribution of root PRR7 to the shoot circadian clock (**Figs. 3**, **5**). We did not notice an obvious association between the accuracy of period length and the amplitude or average period length (**Figs. 3**, **5**, **Supplementary Figs. S4, S8, S11**). Therefore, we propose that the accuracy of period length can be defined as a new parameter of circadian rhythms. In addition to the amplitude, average period length and phase, the estimation of this accuracy is likely important in assessing the robustness of the circadian clock, which may contribute to the precise and timely modulation of physiological events (Abraham et al. 2010, Yan et al. 2021, Greenwood et al. 2022, Wu et al. 2022). Indeed, in rice, changing the day length by 30 min is sufficient to affect flowering-related gene expression levels (Itoh et al. 2010), suggesting that the punctual regulation of gene expression is essential for physiological responses. It will be of great interest to decipher the contribution of timekeeping mechanisms to physiological processes.

## Materials and Methods

### Plant material and growth conditions

The mutant *prr7-3* (Farré et al. 2005), *35S:HA-PRR7* #54 (Farré and Kay 2007), and the transgenic line *LHYpro:LUC* (Torii et al. 2022), both in the *Arabidopsis* (*Arabidopsis thaliana*) accession Col-0, were previously described. A modified growth medium, based on half-strength Murashige and Skoog medium, was described previously and used in this study (Negishi et al. 2018). Except for sucrose treatment, all growth media did not contain any sucrose. The K^+^ concentration in the control growth medium was adjusted to 10 mM. Under low-K^+^ or high-K^+^ conditions, the K^+^ concentration was adjusted to 0.01 or 100 mM, respectively, by the addition of KCl. Under low Mg^2+^ and low Ca^2+^, MgSO_4_ and CaCl_2_ were removed, respectively. For DCMU treatment, the DCMU concentration was adjusted to 2 μM with 20 mM DCMU stock. For sucrose treatment, the sucrose concentration was adjusted to 2% (w/v) with sterilized 20% sucrose stock. Both DCMU and sucrose were added before the medium solidified. As K^+^, Mg^2+^ and Ca^2+^ are provided as KCl, MgSO_4_ and CaCl_2_, the modified medium is also affected in Cl^−^ and SO_4_^2−^ concentrations, which were supplied with HCl and H_2_SO_4_, respectively. All growth media contained 0.8% (w/v) agar, and the pH was adjusted to 6.3 with 1 M Tris-HCl, pH 9.0. Seedlings were grown under cool white fluorescent light (80–100 μmol m^–2^ s^–1^) in a growth chamber (LP-30CCFL-8CTAR-S; NK system) and entrained under a 12-h-light/12-h-dark (LD) photoperiod at 22°C. Except for long-term low-K^+^ conditions, 12-day-old seedlings were transferred to a new growth medium and entrained under LD cycles for another 2 d before being released into LL for LUC imaging. Under long-term low-K^+^ conditions, 7-day-old seedlings were transferred to low-K^+^ medium, entrained under LD cycles for another 9 d and then transferred to LL. Plant images were captured with an iPhone Xs (Apple, Cupertino, California, USA).

### Sucrose quantification

Roots were collected from 30 plants, and the fresh weight was measured. Sugar extraction was previously described (Müller 2016). The sugar pellet was dissolved with 100 µl of H_2_O and measured using a Glucose and Sucrose Assay Kit (BioVision, Milpitas, California, USA).

### RNA extraction and RT-qPCR

Total RNA was extracted from the pooled roots of 30 seedlings using a Maxwell RSC Plant RNA kit (Promega, Madison, Wisconsin, USA) according to the manufacturer’s recommendations. For first-strand cDNA synthesis, 500 ng of total RNA was reverse-transcribed using a PrimeScript RT reagent kit with gDNA Eraser (TaKaRa, Otsu, Japan), and qPCR was performed using THUNDERBIRD SYBR qPCR Mix (TOYOBO, Osaka, Japan) and a CFX96 Real-Time PCR Detection System (Bio-Rad, Hercules, California, USA). The following reaction program was used: pre-denaturation for 30 s at 95°C, followed by 45 cycles of denaturation for 5 s at 95°C, and annealing and elongation for 30 s at 60°C. Data were analyzed by the delta Ct method. *ISOPENTENYL DIPHOSPHATE ISOMERASE2* (*IPP2*) was used as an internal control. A list of primers is provided in **Supplementary Table S1**.

### Measurement of cation transport in xylem sap

We modified the method for xylem sap collection described in the previous study (Tabata et al. 2014, Nakayama et al. 2017). The seedling was cut at the hypocotyl, and the xylem sap was collected by placing a 0.4-mm diameter silicone tube over the hypocotyl stump for 1 h. The xylem sap in the silicon tubes was collected into the microtube by centrifugation. At each time point, xylem sap was collected from 30 plants, and 2 μl of xylem sap was analyzed [Dionex Aquion ion chromatography system (Thermo, Waltham, Massachusetts, USA)].

### Measurement of the transpiration rate

Seedlings were grown on a control medium under LD for 14 d and then transferred to 1:1 soil and vermiculite mixture. For another 14 d, seedlings were entrained under white light (90 μmol m^−2^ s^−1^) with LD at 23°C and then transferred to LL. Stomatal conductance was determined using an LI-6400 portable gas exchange system (LI-COR, Lincoln, Nebraska, USA) equipped with a 6400-15 *Arabidopsis* leaf chamber (LI-COR). The measurements were performed with an airflow of 200 l h^−1^, 350 ppm CO_2_, a relative humidity of 40–60% and 24°C. Data were recorded at 10-s intervals.

### LUC assay

Bioluminescence assays were performed with a Kondotron system (Kondo et al. 1994). The peak time was estimated by fast Fourier transform–nonlinear least squares, and the peak-to-peak period length of four consecutive cycles within a 96-h window under LL was calculated. The average period length was calculated from the mean period length of each seedling. The SD was calculated from the variation in period length observed across individual seedlings. In plotting, the waveforms were smoothed based on kernel smoothing. In addition, to facilitate the comparison of luminescence rhythms from one individual to another, trend removal was performed followed by amplitude trend removal. For K^+^ rescue conditions, the roots were covered with sterile plastic sheets to prevent KCl from coming into contact with the shoots, and a sterile 100 mM KCl solution was poured under the plastic sheets. All growth media contained 250 µM d-luciferin potassium salt (Wako, Osaka, Japan). LUC images were captured with a multifunctional in vivo imaging system (Molecular Devices, San Jose, California, USA).

### Weather data analysis

All historical weather data were obtained from the US National Oceanic and Atmospheric Administration National Center for Environmental Information (NCEI) website (https://www.ncei.noaa.gov/pub/data/uscrn/products/hourly02/2019/). The analysis was carried out using daily data in the spring of 2019 with a day length of 11.5–12.5 h in each city (Cape Charles: 24 d, Durham: 26 d and Redding: 22 d). Water content in the air refers to the volumetric humidity (VH) calculated by the following three equations: RH and *t* represent the relative humidity and temperature, respectively.


$${\mathit{VH}} = a \cdot \frac{{{\mathit{RH}}}}{{100}}$$

$$a = \frac{{217 \cdot e}}{{t + 273.15}}$$

$$e = 6.1078 \cdot {10^{{\displaystyle{\frac{\scriptstyle{7.5t}}{\scriptstyle{t + 237.3}}}}}}$$

The remainder component was decomposed by STL in R version 3.6.2.

### Mathematical analysis

The coupling between shoot and root circadian clocks was modeled with a time delay–coupled system of Stuart–Landau equations subjected to weak noise, as follows:


$$\frac{{\mathit{d}}}{{{\mathit{d}}t}}{z_1}\left( t \right) = {\alpha _1}{z_1}\left( t \right) - {\beta _1}{\left| {{z_1}\left( t \right)} \right|^2}{z_1}\left( t \right) + {k_2}{z_2}\left( {t - {{\Delta }}t} \right) + \sqrt {{D_1}} {\xi _1}\left( t \right)$$

$$\frac{{\mathit{d}}}{{{\mathit{d}}t}}{z_2}\left( t \right) = {\alpha _2}{z_2}\left( t \right) - {\beta _2}{\left| {{z_2}\left( t \right)} \right|^2}{z_2}\left( t \right) + {k_1}{z_1}\left( {t - {{\Delta }}t} \right) + \sqrt {{D_2}} {\xi _2}\left( t \right)$$

where the complex variables ${z_1}\left( t \right)\,$ and ${z_2}\left( t \right)$ represent the dynamics of circadian rhythms in shoots and roots, respectively; ${\xi _1}\left( t \right)$ and ${\xi _2}\left( t \right)$ are independent white Gaussian noise $E\left[ {{\xi _{1,2}}\left( t \right)} \right] = 0$ and $E\left[ {{\xi _{1,2}}\left( t \right){\xi _{1,2}}\left( {t{^{^{\prime}}}} \right)} \right] = \delta \left( {t - t{^{^{\prime}}}} \right)$, where $E\left[ \cdot \right]$ denotes the expectation of time series, and ${k_1}$, ${k_2}$, ${D_1}$ and ${D_2}$ are the connection strength and noise in shoots and roots, respectively. We set ${\alpha _1} = 0.4 + 2i,\,{\beta _1} = 0.4,\,{k_1} = 1.2,\,{D_1} = 0.5,\,{\alpha _2} = 0.1 + 1.9i,\,{\beta _2} = 0.2,\,{k_2} = 0.25,\,{D_2} = 0.35$, assigned 0 for ${k_1}$ and/or ${k_2}$ to model the loss of connection and adjusted the delay (${{\Delta }}t$) to about 1 h. The Euler–Maruyama scheme was used for numerical integration.

### Micrografting

Micrografting was previously described with minor modification (Turnbull et al. 2002). Seedlings were grown on 2.5% agar media without nutrients under LD for 3 d after germination. After the grafting treatment, the grafted plants were incubated for 5 d under LL at 27°C and then transferred to the control medium. Before the LUC assay, the grafted plants were grown under LD at 22°C for another 10 d.

## Supplementary Material

pcad003_Supp

## Data Availability

The climate data are available on the NCEI website (https://www.ncei.noaa.gov/pub/data/uscrn/products/hourly02/2019/). The *Arabidopsis* Genome Initiative numbers for the genes featured in this study are as follows: *DIN6: AT3G47340; SEN5: AT3G15450; BRX1-1: AT3G15460; APUM24: AT3G16810; CCA1: AT2G46830; LHY: AT1G01060; TOC1: AT5G61380; PRR5: AT5G24470; PRR7: AT5G02810; PRR9: AT2G46790; LUX: AT3G46640* and *IPP2: AT3G02780*.
