# Correction To: Root PRR7 Improves the Accuracy of the Shoot Circadian Clock through Nutrient Transport

**DOI:** 10.1093/pcp/pcae026

**Published:** 2024-03-28

This is a correction to: Kyohei Uemoto, Fumito Mori, Shota Yamauchi, Akane Kubota, Nozomu Takahashi, Haruki Egashira, Yumi Kunimoto, Takashi Araki, Atsushi Takemiya, Hiroshi Ito, Motomu Endo, Root PRR7 Improves the Accuracy of the Shoot Circadian Clock through Nutrient Transport, Plant and Cell Physiology, Volume 64, Issue 3, March 2023, Pages 352–362, https://doi.org/10.1093/pcp/pcad003

The original published version of this article contained factual inaccuracies in the description of previous related work in the field. In the interest of ensuring accuracy and removing any bias in the scientific record, the following section of the introduction should be corrected from:

‘Unlike the mammal circadian clock with a central oscillator residing in the suprachiasmatic nucleus that controls circadian rhythms in peripheral organs, the plant circadian clock shows no obvious master clock, instead forming a decentralized and complex network architecture (Shimizu et al. 2015)’.

to:

‘In mammals, circadian rhythms are governed by a central oscillator residing in the suprachiasmatic nucleus, which controls circadian rhythms in peripheral organs. In plants, a hierarchical communication framework has been proposed, wherein the root clock relies on a long-distance signal from the shoot apex (Takahashi et al., 2015), and at the same time the leaf tissues contribute to a decentralized network architecture (Shimizu et al., 2015)’.

