# Supplementary material for: Root PRR7 Improves the Accuracy of the Shoot Circadian Clock through Nutrient Transport
Source: Plant Cell Physiol. 2023 Jan 7;64(3):352–62. doi: 10.1093/pcp/pcad003 (PMC10016326; doi:10.1093/pcp/pcad003)
Supplement: pcad003_Supp [file pcad003_supp.zip › suppl_data/pcp-2022-e-00289-File007.pdf]

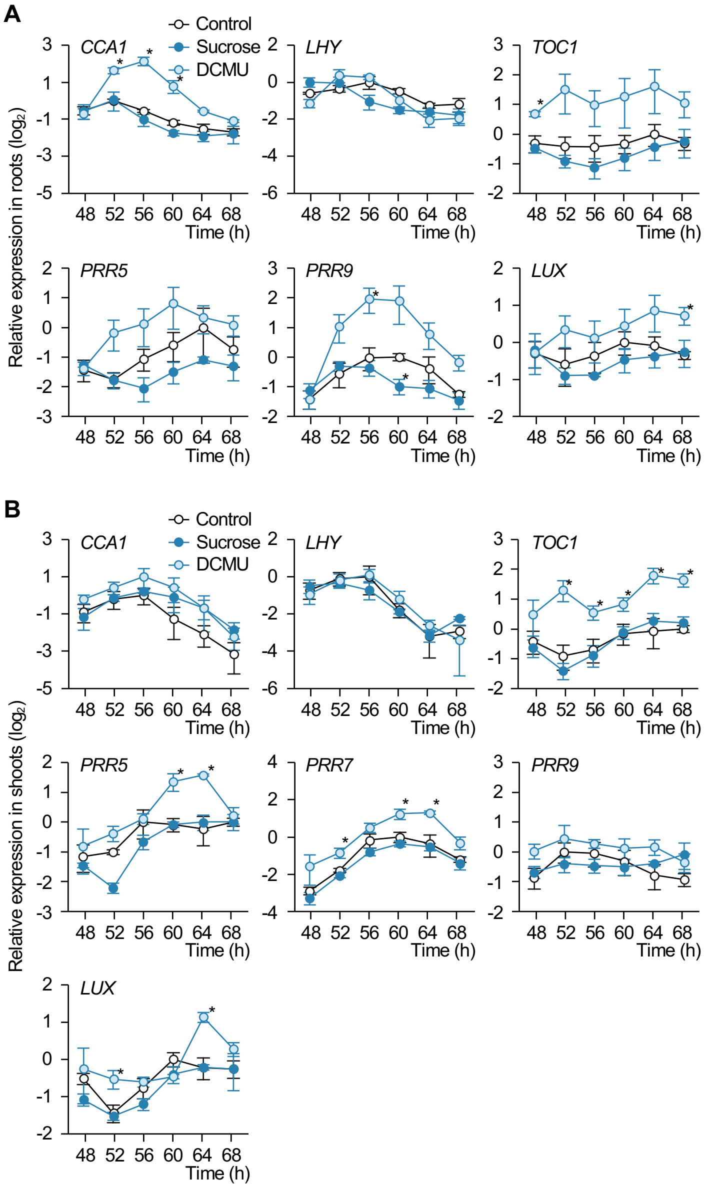

Supplemental Figure. S1. Sucrose affects the expression levels of clock genes in roots and shoots. **(A, B)** Time course of clock genes expression by RT-qPCR in WT roots **(A)** or in WT shoots **(B)** with exogenous sucrose or DCMU (n =3). Mean ± SEM. *P < 0.05 compared to Control; Dunnett's test **(A, B)**.
