# Supplementary material for: Root PRR7 Improves the Accuracy of the Shoot Circadian Clock through Nutrient Transport
Source: Plant Cell Physiol. 2023 Jan 7;64(3):352–62. doi: 10.1093/pcp/pcad003 (PMC10016326; doi:10.1093/pcp/pcad003)
Supplement: pcad003_Supp [file pcad003_supp.zip › suppl_data/pcp-2022-e-00289-File008.pdf]

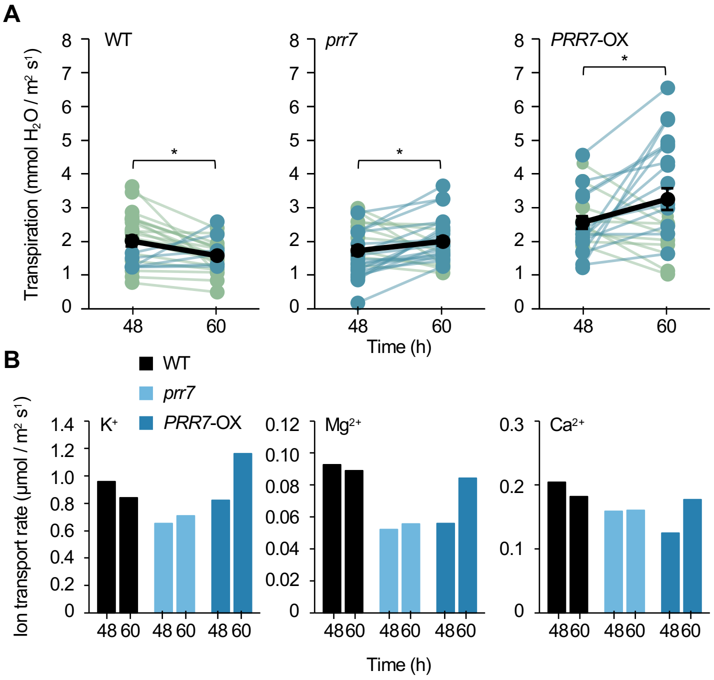

Supplemental Figure. S2. Root PRR7 is involved in the regulation of K^+ contents transported to shoots.

(A) The transpiration rate in WT, *prr7*, and *PRR7*-OX (WT; $n = 24$, *prr7*; $n = 27$, *PRR7*-OX; $n = 23$). Each line represents biological replicates. Green lines show the decreasing trend, and blue lines show the increasing trend. Black lines show the average of transpiration rate. Mean ± SEM. *$P < 0.05$ compared to Time 48; two-sided Student's t-test **(B)** The cation transport rate calculated from the transpiration rate and cation concentration of xylem sap.
