# Supplementary material for: Root PRR7 Improves the Accuracy of the Shoot Circadian Clock through Nutrient Transport
Source: Plant Cell Physiol. 2023 Jan 7;64(3):352–62. doi: 10.1093/pcp/pcad003 (PMC10016326; doi:10.1093/pcp/pcad003)
Supplement: pcad003_Supp [file pcad003_supp.zip › suppl_data/pcp-2022-e-00289-File009.pdf]

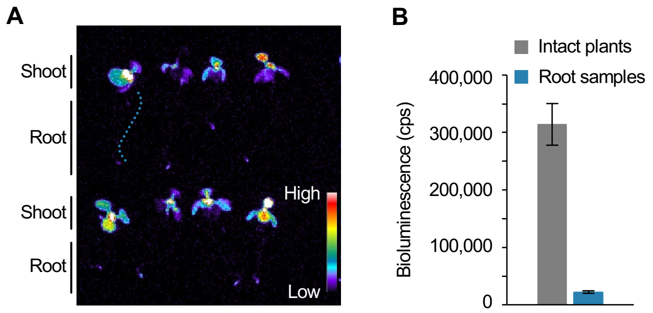

Supplemental Figure. S3. ***LHYpro:LUC*** **bioluminescence from intact plants is mainly derived from shoots.**

LHYpro:LUC bioluminescence in intact plants and root samples at *Zeitgeber Time* 2 when *LHY* promoter activity is maximized. **(A)** Pseudo color representation of the bioluminescence of *LHYpro:LUC* seedlings under control condition. Broken blue line indicates the root of the upper left plant. **(B)** Bioluminescence of *LHYpro:LUC* (n = 5). Mean ± SEM. cps: counts per second.
