# Supplementary material for: Root PRR7 Improves the Accuracy of the Shoot Circadian Clock through Nutrient Transport
Source: Plant Cell Physiol. 2023 Jan 7;64(3):352–62. doi: 10.1093/pcp/pcad003 (PMC10016326; doi:10.1093/pcp/pcad003)
Supplement: pcad003_Supp [file pcad003_supp.zip › suppl_data/pcp-2022-e-00289-File010.pdf]

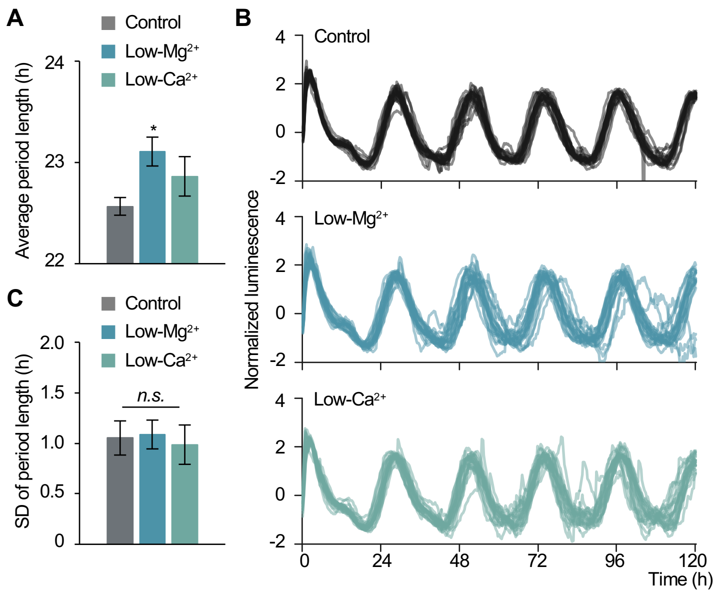

Supplemental Figure. S4. Neither low-Mg^{2+} nor low-Ca^{2+} affects the accuracy of period length in shoots.

Circadian oscillations of *LHYpro:LUC* under control, low-Mg^{2+}, or low-Ca^{2+} conditions in LL (n = 20). **(A)** Average period length, as calculated for each plant across the four days in LL. **(B)** Waveforms of individual *LHYpro:LUC* bioluminescence. Waveforms were detrended and compensated for decreasing amplitude over the time course. **(C)** Standard deviation (SD) of period length, as calculated for each plant across the four days in LL. Data are means ± SEM. * $P < 0.05$ compared to control by Dunnett's test.
