# Supplementary material for: Root PRR7 Improves the Accuracy of the Shoot Circadian Clock through Nutrient Transport
Source: Plant Cell Physiol. 2023 Jan 7;64(3):352–62. doi: 10.1093/pcp/pcad003 (PMC10016326; doi:10.1093/pcp/pcad003)
Supplement: pcad003_Supp [file pcad003_supp.zip › suppl_data/pcp-2022-e-00289-File011.pdf]

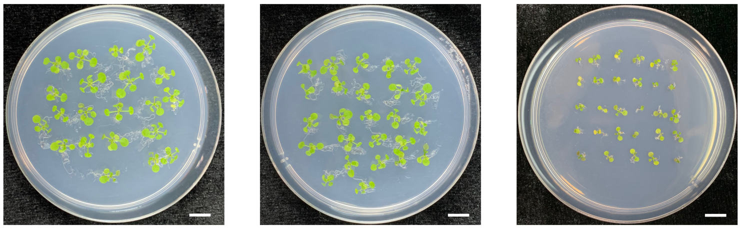

Supplemental Figure S5. Our low-K^+ condition does not cause the severe phenotypic defects.

The image was captured at time 72 under LL. WT plants were grown in control medium (10 mM K^+) under LD for 14 days, and then transplanted to either control or low-K^+ medium. After transplanting, plants were entrained in LD cycle for another 2 days and then transferred under LL condition. Long-term low-K^+ plants were grown in control for 7 days, and then grown in low-K^+ for 9 days before LL condition started. Scale bars represent 1 cm.
