# Supplementary material for: Root PRR7 Improves the Accuracy of the Shoot Circadian Clock through Nutrient Transport
Source: Plant Cell Physiol. 2023 Jan 7;64(3):352–62. doi: 10.1093/pcp/pcad003 (PMC10016326; doi:10.1093/pcp/pcad003)
Supplement: pcad003_Supp [file pcad003_supp.zip › suppl_data/pcp-2022-e-00289-File014.pdf]

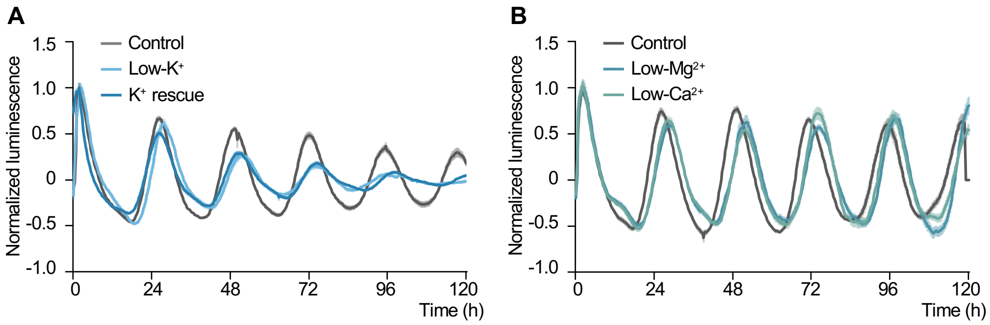

Supplemental Figure S8. K^+ resupply does not neutralize the effect of amplitude or average period length in shoots.

(A, B) Circadian oscillations of *LHYpro:LUC* under control, low-K^+ or K^+ rescue conditions **(A)** and under low-Mg^{2+} or low-Ca^{2+} **(B)** in LL (n = 20). Waveforms indicates the mean of *LHYpro:LUC* bioluminescence in each conditions. Waveforms are detrended but not corrected for amplitude. Data are means ± SEM.
