# Supplementary material for: Root PRR7 Improves the Accuracy of the Shoot Circadian Clock through Nutrient Transport
Source: Plant Cell Physiol. 2023 Jan 7;64(3):352–62. doi: 10.1093/pcp/pcad003 (PMC10016326; doi:10.1093/pcp/pcad003)
Supplement: pcad003_Supp [file pcad003_supp.zip › suppl_data/pcp-2022-e-00289-File015.pdf]

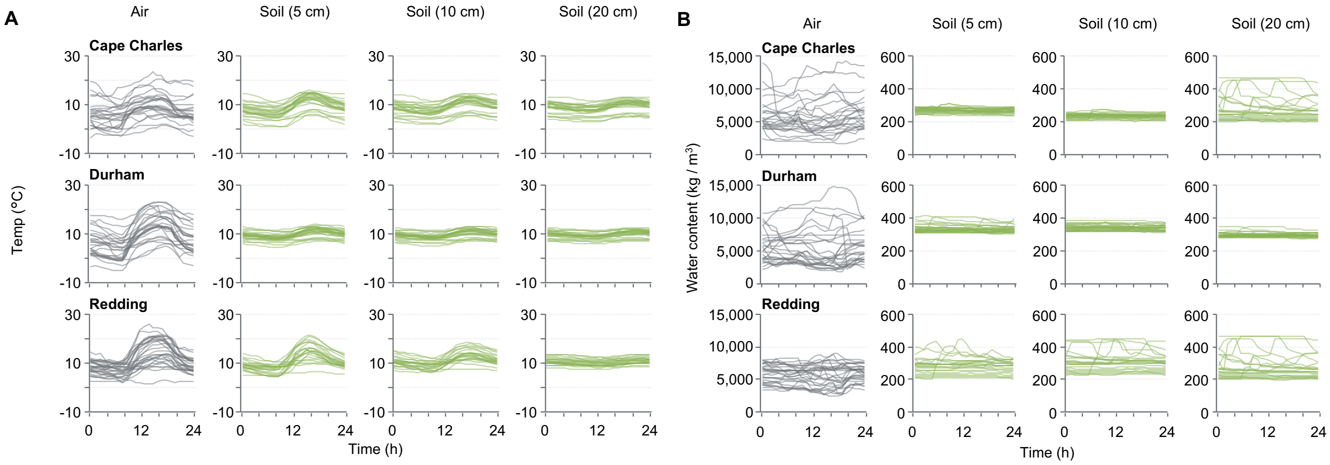

Supplemental Figure S9. Climate data from Cape Charles, Durham, and Redding in the United States.

Climate data for ambient and soil temperature **(A)**, and for humidity and soil moisture **(B)** in the spring of 2019 at Cape Charles, Durham, and Redding in the United States (Cape Charles; n = 24, Durham; n = 26, Redding; n = 22).
