# Supplementary material for: Root PRR7 Improves the Accuracy of the Shoot Circadian Clock through Nutrient Transport
Source: Plant Cell Physiol. 2023 Jan 7;64(3):352–62. doi: 10.1093/pcp/pcad003 (PMC10016326; doi:10.1093/pcp/pcad003)
Supplement: pcad003_Supp [file pcad003_supp.zip › suppl_data/pcp-2022-e-00289-File016.pdf]

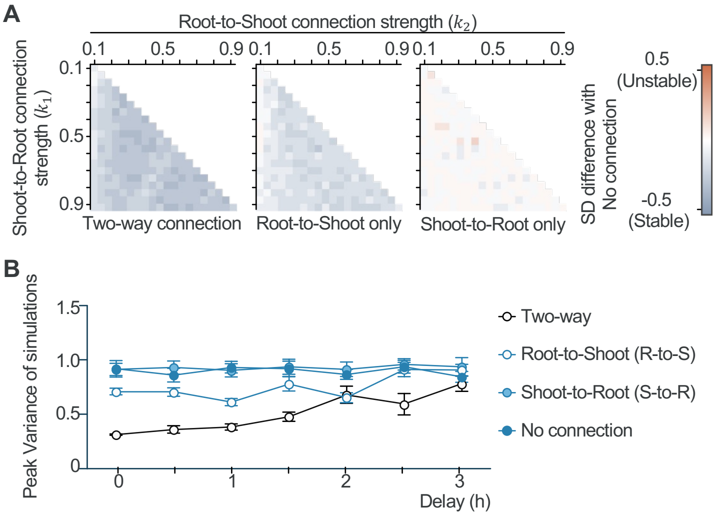

Supplemental Figure. S10. Grid search of parameters of connection strength and time-lag.

(A) The difference in the SD of period length compared to "No connection" in each condition when the magnitude of k_1 and k_2 is varied from 0.1 to 0.9 and k_1 is higher than k_2 ($k1 > k2$). (n = 50). **(B)** The SD of period length in Shoot Oscillator when the time lag is varied from 0 h to 3 h under each condition (n = 50). Mean ± SEM.
