# Supplementary material for: Root PRR7 Improves the Accuracy of the Shoot Circadian Clock through Nutrient Transport
Source: Plant Cell Physiol. 2023 Jan 7;64(3):352–62. doi: 10.1093/pcp/pcad003 (PMC10016326; doi:10.1093/pcp/pcad003)
Supplement: pcad003_Supp [file pcad003_supp.zip › suppl_data/pcp-2022-e-00289-File017.pdf]

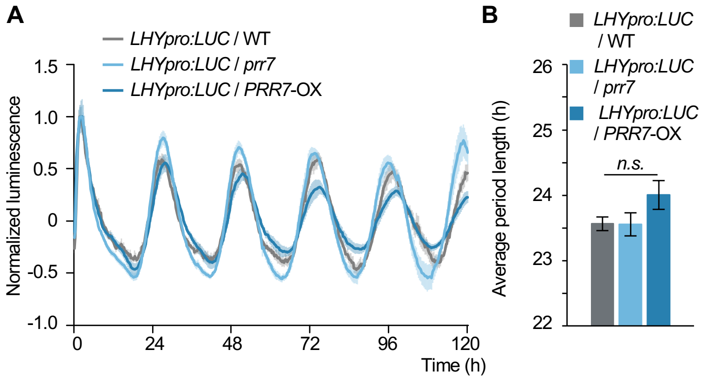

Supplemental Figure. S11. Root PRR7 does not affect the amplitude and average period length of shoot circadian rhythm.

LHYpro:LUC scions were grafted onto WT (*LHYpro:LUC*/WT), *prr7* (*LHYpro:LUC*/*prr7*), or *PRR7*-OX (*LHYpro:LUC*/*PRR7*-OX) rootstocks. (A) Circadian oscillations of *LHYpro:LUC* in LL. **(A, B)** The mean of *LHYpro:LUC* bioluminescence traces **(A)** and mean of period length under LL (n = 15) **(B)**. **(A)** Waveforms were detrended, not compensated for the decrease in amplitude, over the time course. Data are means ± SEM. * P < 0.05 compared to *LHYpro:LUC/WT* by Dunnett's test.
