# Supplementary material for: Root PRR7 Improves the Accuracy of the Shoot Circadian Clock through Nutrient Transport
Source: Plant Cell Physiol. 2023 Jan 7;64(3):352–62. doi: 10.1093/pcp/pcad003 (PMC10016326; doi:10.1093/pcp/pcad003)
Supplement: pcad003_Supp [file pcad003_supp.zip › suppl_data/pcp-2022-e-00289-File018.pdf]

Table S1: List of primers used in this study.

Target gene	primer1	primer2	Reference
IPP2	GTATGAGTTGCTTCTCCAGCAAAG	GAGGATGGCTGCAACAAGTGT	Shimizu et al., 2015
DIN6	AACTTGTCGCCAGATCAAGG	GGAACACGTGCCTCTAGTCC	Baena-González et al., 2007
SEN5	GCGAAACTCTCTCCGACTTC	CCACAGAACAACCTTTGACG	Rodrigues et al., 2013
APUM24	GCTTGTCAACAGTGGCCTTG	TCACACAGCTTCTCGCTCAG	Maekawa et al., 2018
BRX1-1	GAATGATAAAAGTCGACCAAAGTTTC	CTTCTTCGTGACATAGCAGTCTTTAC	Maekawa et al., 2018
CCA1	GAGGCTTTATGGTAGAGCATGGCA	TCAGCCTCTTTCTCTACCTTGGAGA	Endo et al., 2014
LHY	GGTTCTGTACCATATTACCC	GTAGCAGATGACATATAGACC	
TOC1	GCCTCTTCGCACCAACGAGCT	TCAGCAAGTCCTAGCATGCGTCT	Endo et al., 2014
PRR5	ATTCCGAATGAAGCGAAAGGA	TCGTAACGAACCTTTTTCTCATAACAT	Mockler et al., 2004
PRR7	AGGTGCTTCCGAAAGAAGGTACGA	TGGGCTGAGAAATAGTGGGTTTTGT	Frank et al, 2018
PRR9	TGCTTTGACAAAAAGGTTCGGTACCAGAGCAGGA	ACGCGTCTGAATTCACGGTTCGCACG	
LUX	ATCATGGAGCTGGTGGAAATGG	TGCAATTTGGGACTTTGCGGT	
